# LIDAR-based characterization and conservation of the first theropod dinosaur trackways from Arkansas, USA

**DOI:** 10.1371/journal.pone.0190527

**Published:** 2018-01-02

**Authors:** Brian F. Platt, Celina A. Suarez, Stephen K. Boss, Malcolm Williamson, Jackson Cothren, Jo Ann C. Kvamme

**Affiliations:** 1 Department of Geology and Geological Engineering, University of Mississippi, University, Mississippi, United States of America; 2 Department of Geosciences, University of Arkansas, Fayetteville, Arkansas, United States of America; 3 Center for Advanced Spatial Technologies, University of Arkansas, Fayetteville, Arkansas, United States of America; Perot Museum of Nature and Science, UNITED STATES

## Abstract

LIDAR-based analyses of the first theropod dinosaur trackways known from the state of Arkansas, USA are reported. The trackways were found on a limestone bedding plane in the Albian De Queen Formation in an active gypsum quarry. Because limited access precluded thorough field study, fieldwork focused on preserving the entire site digitally with ground-based LIDAR, and detailed measurements were later taken digitally from point cloud data. The site contains eight tridactyl trackways associated with sauropod trackways and numerous isolated tracks. Although there appear to be two different tridactyl morphotypes, we show that the tracks are all likely from a single species of trackmaker. We apply a simple method of estimating substrate consistency by comparing the differences between true track dimensions and apparent track dimensions. The tridactyl tracks at the southern end of the site are preserved with significantly greater differences in true *vs*. apparent dimensions and are shallower than the rest of the tridactyl tracks at the site, which we interpret as the result of outward expansion of the soft tissues of the foot upon contact with a firm substrate. We interpret the firm substrate as having high bulk density and high shear strength, which also explain associated manus-only sauropod tracks. We show that the tridactyl tracks are likely from theropod trackmakers and that footprint lengths, trackway paces, stride lengths, and pace angulations of the De Queen trackways are statistically indistinguishable from equivalent measurements of theropod trackways in the Glen Rose Formation. The Glen Rose tracks are attributed to the large-bodied theropod, *Acrocanthosaurus* and we likewise attribute the De Queen tracks to *Acrocanthosaurus*, which is known from skeletal remains in temporally equivalent units and from the mine itself.

## Introduction

**Fig 1:**
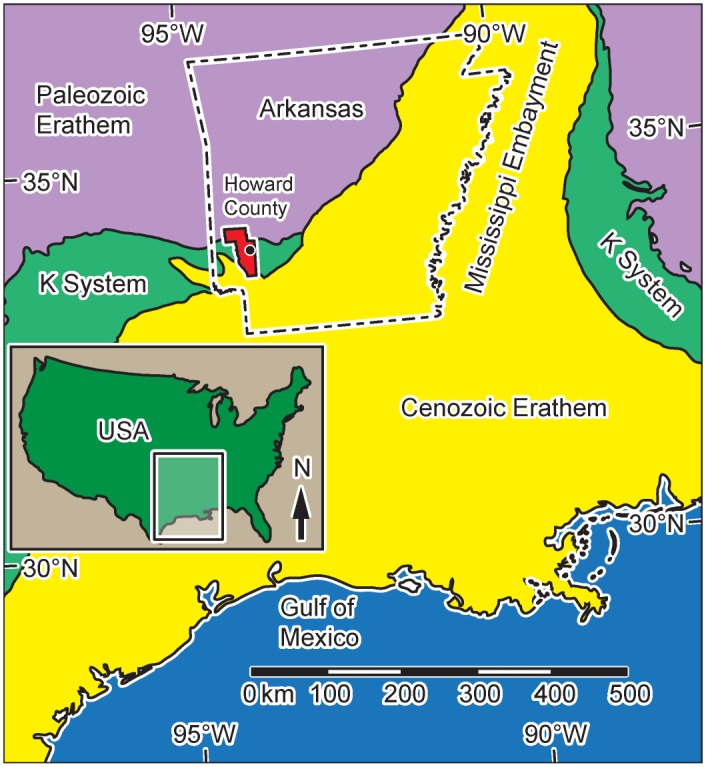
Simplified geologic map of the western US Gulf Coast. Note surface expression of the Cretaceous System and the location of Howard County, Arkansas. Black circle represents the location of the gypsum mine in Howard County. Blue = modern Gulf of Mexico, yellow = Cenozoic strata, green = Cretaceous strata, and purple = Paleozoic strata.

Dinosaur trackways of the United States Gulf Coastal Plain have received much attention since discoveries of large sauropod and theropod tracks in the Cretaceous Glen Rose Formation of Texas in the 1930s [1]. Numerous sites containing sauropod, theropod, and ornithopod trackways are now known from shoreline and carbonate mudflat facies within the Cretaceous System, which is exposed in the Edwards Plateau and Lampasas Cut Plain regions of Texas and along an arcuate band that passes through southeastern Oklahoma and pinches out along the southwestern edge of the Mississippi Embayment in western Arkansas (Fig 1). Dinosaur tracksites in these strata were known only from Texas until the 1983 discovery of hundreds of sauropod tracks at the Briar Site, a gypsum quarry in Howard County, Arkansas (Fig 1) [2]. The recent discovery of tridactyl tracks associated with sauropod tracks near the original Briar Site, however, has changed our perspective of the paleoecology of this area [3]. Here we report LIDAR-based characterization of the new site, including analysis of the first known theropod dinosaur tracks in the state of Arkansas, as well as a basic approach to evaluating substrate consistency.

The tracks described herein were found in the actively mined area of the CertainTeed Gypsum mine and the science team was granted only a small window of time to document the site. The time constraints and large areal extent of the track-bearing surface (approximately 4,200 m^2^) [3] precluded fieldwork focused on detailed measurements and casting of every track and trackway. Furthermore, highly dinoturbated areas of the track-bearing surface hampered efforts to isolate and follow individual trackways through observations at ground level. Priority, therefore, was given to digital preservation of the site for future, multi-scale observations and data analyses. The purpose of this paper is to report the results of those efforts.

## Previous work

LIDAR has become an increasingly common tool for preserving significant or endangered dinosaur tracksites. It was first successfully integrated with photogrammetry and GIS at the Middle Jurassic Red Gulch Dinosaur Tracksite in Wyoming to make a high-resolution digital terrain model that preserved the site, which is open to the public and is vulnerable to weathering and vandalism [4]. Similarly threatened, the Late Cretaceous Fumanya dinosaur tracksites in the Pyrenees of Catalonia were documented using LIDAR because of significant erosion enhanced by steeply sloping track-bearing strata, which also limited physical access to the tracks [5,6]. Digital outcrop models of those sites were created before any additional erosion could occur, while also offering the opportunity to perform detailed analyses and comparisons of the tracks [5–7]. The Late Jurassic Chevenez-Combe Ronde tracksite in the Canton of Jura, NW Switzerland was threatened by highway construction and preserved at high resolution with LIDAR and photogrammetry [8]. The dinosaur tracksites of the Lower Cretaceous Glen Rose Formation in the Paluxy River of Texas are significant for being the first and some of the best examples of sauropod tracks discovered [1], containing potential evidence of interaction between a theropod and a sauropod [9,10], and preserving the material that serves as the type for *Brontopodus birdi* [11,12]. LIDAR was combined with GIS, photography, tracings, measurements, and historical documentation to preserve many of these tracks, including sections of a trackway that were excavated and are housed in museums, where some deterioration is occurring [13]. The Las Cerradicas tracksite in Galve, Teruel, Spain contains some of the oldest quadrupedal ornithopod trackways and LIDAR was used with photogrammetry to discern fine-scale topographic differences in the track surface [14]. Finally, tracksites in the Lower Cretaceous Broome Sandstone in Minyirr, Western Australia contain the type material of *Megalosauropus broomensis* [15] and are threatened by tourism and their location along the coast in an intertidal zone [16]. The location was used to test multiple means of remote sensing techniques, including LIDAR, to evaluate feasibility for capturing data from additional Broome Sandstone tracksites with limited access [16]. Given the variety of preservational techniques available for geoconservation, we used LIDAR, photographs, field measurements, and plaster casts to preserve a significant tracksite threatened by imminent destruction by mining operations.

## Geologic setting, location, and methods

The Albian De Queen Formation is part of the Lower Cretaceous Trinity Group and is composed mainly of bedded gypsum and interbedded and mixed clastics and carbonates deposited in a low-gradient coastal sabkha-tidal flat complex in the ancestral Gulf of Mexico south of the weathered Ouachita highlands [2,17,18]. The previously known De Queen sauropod tracks were found at two stratigraphic intervals, including a laterally extensive, track-bearing limestone bedding plane in the upper part of the formation that was interpreted as a broad carbonate mudflat environment equivalent to the Glen Rose Formation of Texas [2,17].

The new tridactyl tracks were discovered by workers at the CertainTeed Gypsum mine, near the town of Nashville, AR. The mine was formerly operated by Weyerhaeuser, when its quarry became known as the Briar Site [2], although the tracks we describe are from a different area of the mine than the previously described sauropod tracks. The approximate bounding coordinates of the mine property are 34° 05' 09" N, 93° 54' 45" W; 34° 05' 09" N, 93° 50' 11" W; 34° 03' 21" N, 93 50' 11" W; and 34° 03' 21", N 93 54' 45" W.

Permission to access the site was granted by the Director of Operations and physical access was not allowed until all scientific team members had completed Mine Safety and Health Administration (MSHA) compliance training. While on site, investigators were required to have identification and documentation of MSHA compliance training completion on their person and to wear appropriate personal protective equipment at all times.

Excavation of overburden down to the track-bearing limestone was accomplished by blasting and removal with heavy equipment. Initial cleaning of the track-bearing bedding plane utilized a high-pressure compressed air jet and tractor-mounted sweeper, followed by loose sediment removal with brooms and brushes. Initial documentation was performed by constructing a 1 m x 1 m square grid over the track-bearing surface, photographing each grid square, and locating the tracks using distance and azimuth from a datum. Grid photographs were orthorectified and compiled into a photomosaic of the surface. Additional overhead photographs were taken at several perspectives from atop a mobile boom lift. LIDAR data were collected with a Z+F IMAGER^®^ 5006i laser scanner mounted, inverted, on a boom lift and a tripod-mounted Leica ScanStation C10. Unfortunately, boom-lift photography followed heavy rains, which left many tracks full of water; this precluded photogrammetry because water levels would have provided false track bottom surfaces. The Leica ScanStation C10 was chosen because its green laser was able to penetrate the water to record actual track floors.

We established a track-labeling scheme for continuity, where the prefix BT was used for bipedal tridactyl trackways and the prefix Q was used for quadrupedal trackways. Each type of trackway was numbered consecutively from southwest to northeast. Individual tracks within trackways were numbered consecutively in the direction of travel, beginning with the first exposed track.

**Fig 2:**
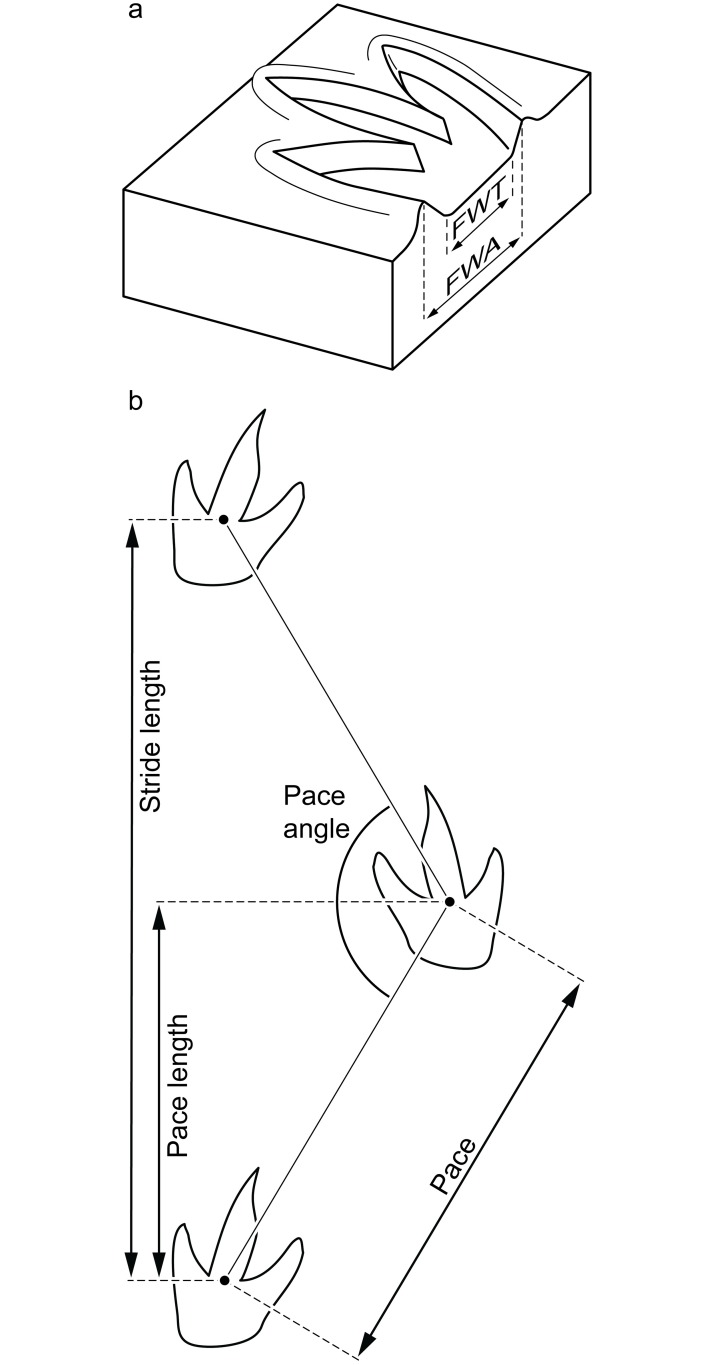
Examples of track and trackways measurements. (a) True versus apparent track dimensions. Note that this example shows the difference between true and apparent width through an arbitrary cross-section of a track, while we measured true and apparent foot width at the point of maximum track width. (b) Trackway measurement conventions and terminology followed in this study.

**Fig 3:**
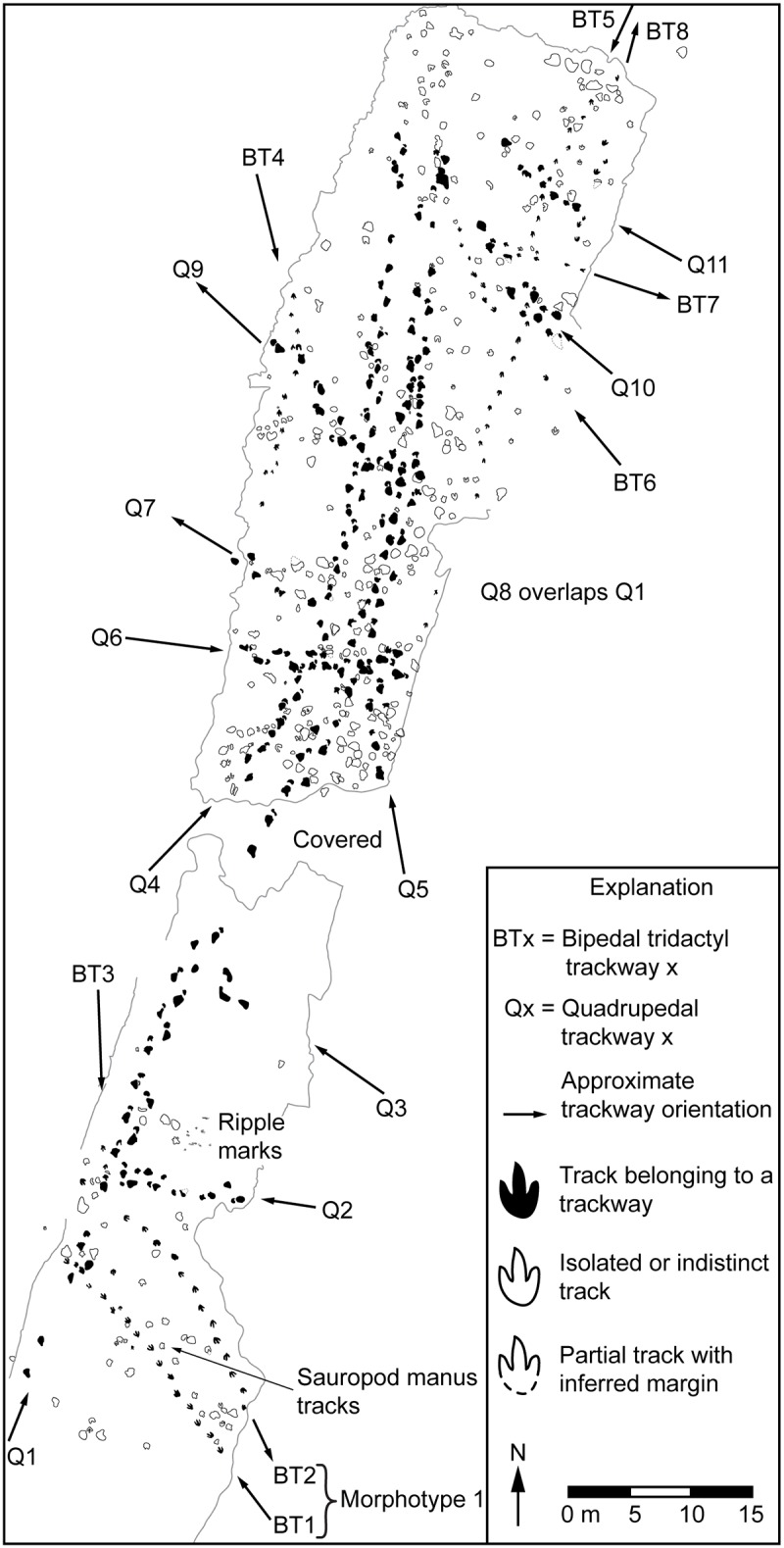
Tracksite map. Illustration of all tracks and trackways exposed at the site. See S1 Fig for high-resolution images and additional detail.

Track and trackway measurements were taken according to previously established standards [19–23]. Track measurements include true foot length (FLT) and true foot width (FWT), which are measured along the track floor and provide the dimensions of the portion of the foot that was in contact with the ground [22] (Fig 2a). Apparent foot length (FLA) and apparent foot width (FWA) were measured where the track shaft walls intersect the tracking surface [22] (Fig 2a). We also took measurements of digit lengths and widths, angles of divarication between individual digits, total divarication, and, in some cases, maximum track depth. Trackway measurements taken include pace, pace length, stride length, and pace angle (Fig 2b). Plaster casts were taken of the best-preserved tracks. Because the casts would be the only physical records of the tracks, we chose to use plaster for its wide availability, high fidelity, very low expansion coefficient [24], and high rigidity upon curing. Plaster was also the most inert and nonhazardous of available casting media according to a comparison of material safety data sheets, so there would be no associated liability with using it on mine property. Also, the flexibility of silicone or latex lends itself to potential distortion, requiring extra materials to provide a rigid backing and latex, in particular, is subject to shrinkage [24]. Tracks did not exhibit overhangs so there were no issues with cast removal that would require a flexible casting medium. Severe time constraints limited the number of measurements that could be taken in the field so the majority of measurements were taken from LIDAR data using Leica Cyclone software. Careful review of field notes, LIDAR data, the photomosaic, and overhead photography allowed construction of a map of all tracks exposed at the site (Fig 3; S1 Fig). All statistical analyses of track measurements were performed in R unless otherwise noted.

In addition to documentation of the track-bearing surface, we measured a detailed stratigraphic section bracketing the track bed to place it in stratigraphic context. The thickness of the measured section was limited by the thicknesses of exposures that could be accessed safely, as determined by the mine safety officer.

## Results and discussion

### Stratigraphic context

**Fig 4:**
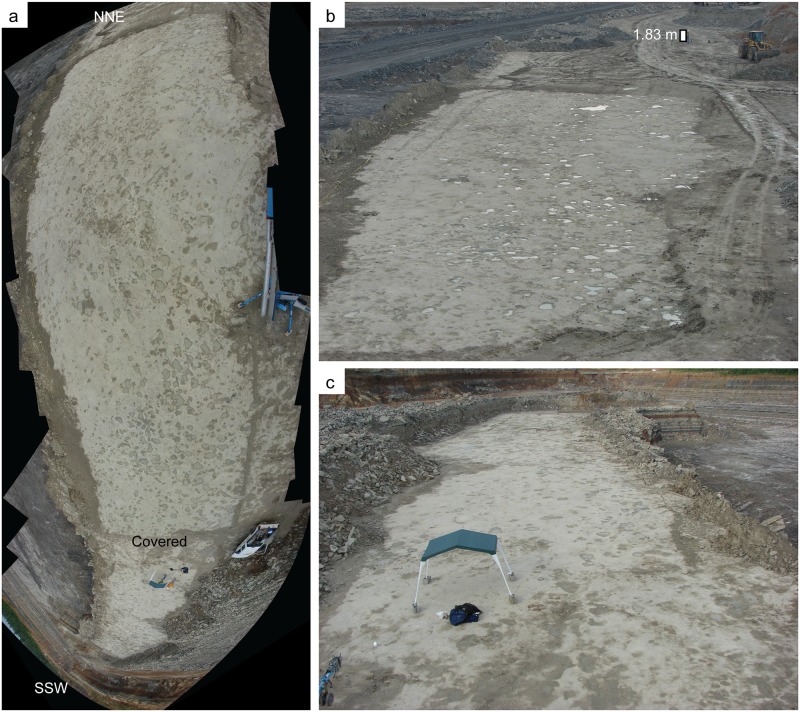
Overview of the track-bearing surface. (a) Composite panoramic image from NNE to SSW showing the entire track-bearing surface, albeit with considerable distortion. Image made with Microsoft Image Composite Editor (ICE). Pickup truck for scale; (b) Oblique view of NNE portion of tracksite; reflective water fills and highlights many tracks. Image taken from boom lift. Person for scale is 1.83 m tall. (c) Oblique view of SSW portion of tracksite; image taken from boom lift. Concrete blocks at base of tent are 20 cm wide and 41 cm long.

**Fig 5:**
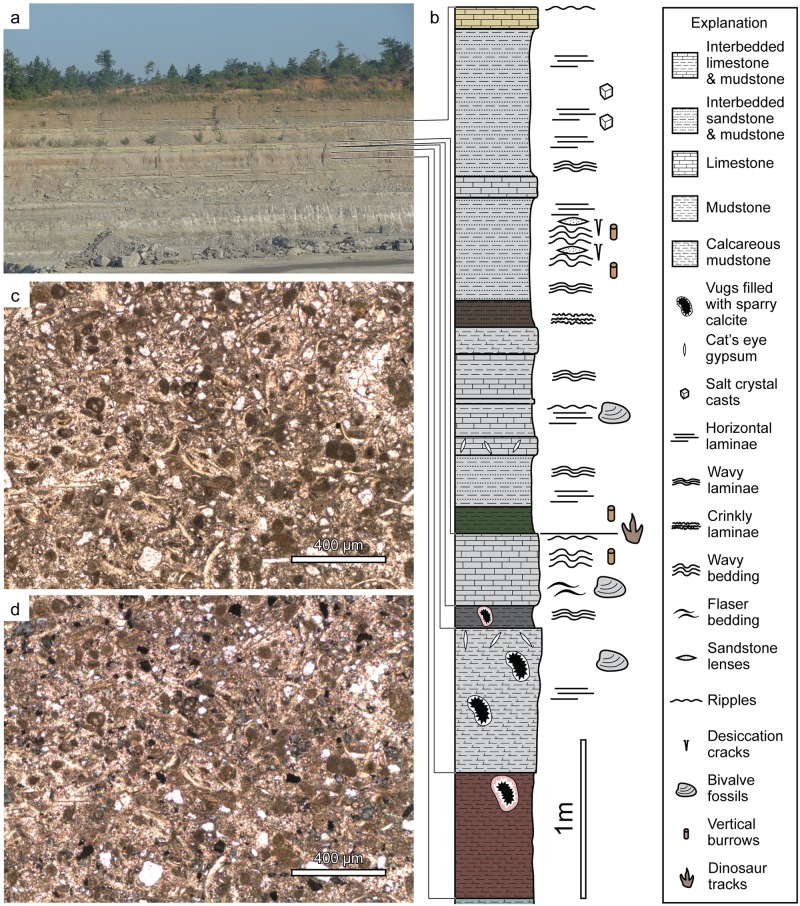
Stratigraphic context of tracksite. (a) Mine high wall showing stratigraphy. (b) Measured section bracketing the track-bearing bed. (c) Photomicrograph of thin section of the track-bearing bed.

The tracks (Fig 3) are preserved on the upper bedding plane (Fig 4) of a ~0.5 m-thick, resistant, massive to thickly bedded, gray limestone bed within a section of massive calcareous shales, carbonate mudstones, and heterolithic intervals consisting of interbedded sandstone and shale (Fig 5A). The shales and carbonate mudstones are locally laminated and contain sporadic marine invertebrate macrofossils and likely represent lagoon and low-energy subtidal to offshore marine environments. The heterolithic intervals contain abundant wavy, lenticular, and flaser bedding, which indicate strong tidal influence within intertidal and shallow subtidal environments. Desiccation cracks, salt crystal casts, and gypsum throughout the section indicate multiple episodes of subaerial exposure in supratidal to shallow intertidal settings on mudflats in a highly evaporative setting.

The track-bearing bed is a biopeldismicrite or biopelmicrite with sparry to microsparry regions and 25–50% subangular quartz grains (Fig 5B). Fossil allochems include thin-shelled bivalves, small gastropods, and foraminifera. The track-bearing bed contains locally abundant low-wavelength, symmetrical ripples.

Immediately overlying the track-bearing bed is a thin, green mudstone with mm-scale diameter, sand-filled vertical burrows (*Skolithos*). The green shale represents a landward shift in facies, making the track-bearing bedding plane a marine flooding surface. The lithology of the track-bearing bed, and its contact with the overlying green mudstone lead us to correlate the track surface described herein to the upper track bed of the previously described Briar Site [2].

### Tridactyl track morphotypes

**Fig 6:**
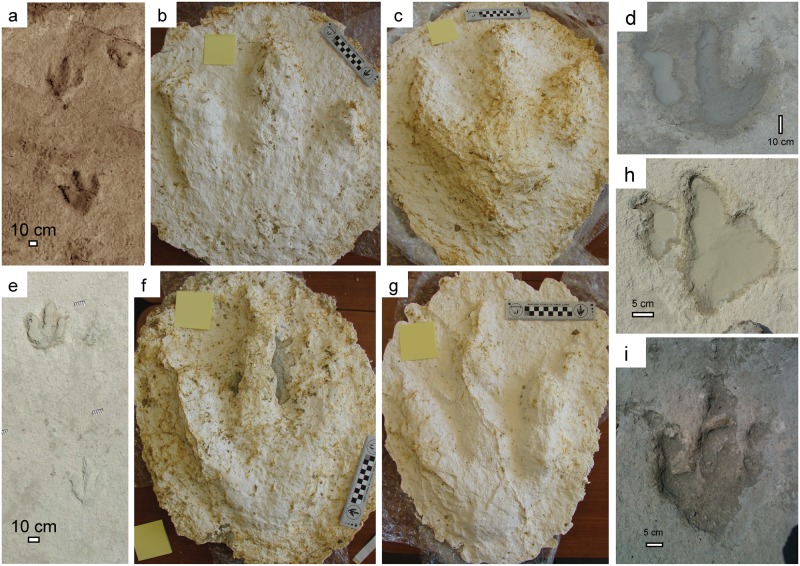
Tridactyl track morphotypes present at the site. (a) two consecutive morphotype 1 tracks from photomosaic; (b) plaster cast of morphotype 1 track, note claw impressions; (c) plaster cast of morphotype 1 track, note blunt “heel” impression; (d) morphotype 1 track filled with mud from recent rain, highlighting “detached” digit II; (e) two consecutive morphotype 2 tracks; (f) plaster cast of morphotype 2 track, note claw impressions and phalangeal pad impressions highlighted by matrix adhering to digit III; (g) plaster cast of morphotype 2 track, note claw impressions and phalangeal pad impressions; (h) morphotype 2 track filled with water, highlighting “detached” digit II; (i) morphotype 2 track showing curved digits, phalangeal pad impressions, and ridges possibly resulting from soft mud flowing into footprint following foot removal.

Upon initial assessment, two tridactyl morphotypes were present. Morphotype 1 tracks (Fig 6a–6d) are 44 to 68 cm long and 33 to 56 cm wide (true dimensions) with blunt to rounded and pointed “heels”, wide, robust, blunt toes, digit III longest, and digit II nearly equal to or shorter than digit IV. Many digit II impressions are detached, i.e., connected to digit III at an elevation above the track floor, whereas digits III and IV intersect at the track floor (Fig 6d). The average angle of divarication between digits II and IV is 33°. Blunt, curved claw impressions are present on some digits and phalangeal pad impressions are rare. This track type is only found in two trackways at the southern end of the tracksite (BT1 and BT2; Fig 3, S1 Fig).

Morphotype 2 tracks (Fig 6e–6i) are smaller, 31 to 58 cm long and 28 to 51 cm wide with straight to curved, mostly more slender digits, pointed heels or no heels, digit III the longest, with digit II nearly equal to or shorter than digit IV. In some examples, digit II is “detached” from digits III and IV (Fig 6h). The average angle of divarication between digits II and IV is 37°. Many digits contain curved claw impressions and few examples of phalangeal pad impressions are preserved.

### Tridactyl trackway measurements

Two morphotype 1 trackways and six morphotype 2 trackways are visible at the site (Fig 3; S1 Fig) and both morphotypes are associated with sauropod tracks identical to those reported previously from the mine [2]. The exposed tridactyl trackways vary in length and several include examples of poorly preserved individual tracks and tracks that remained covered with overburden. Accounting for those tracks, the exposed morphotype 1 trackways each contain 15 tracks, and the morphotype 2 trackways range from six tracks to ~32 tracks in length.

Morphotype 1 trackway paces range between 132 and 327 cm, with a mean of 168 cm. Pace lengths of morphotype 1 trackways vary from 132.2 to 324.3 cm, with a mean of 160.3 cm. Morphotype 1 stride lengths vary between 278.3 and 347.9 cm, with a mean of 308.1 cm. Pace angles for these trackways rage from 164.9° to 177.2°, with a mean of 160.4°.

The paces of morphotype 2 trackways range from 112.7 to 270.6 cm, with a mean of 150.9 cm. Morphotype 2 pace lengths vary from 107.5 to 265.4 cm, with a mean of 145.8. Stride lengths range from 221.5 to 430.7 cm, with a mean of 293.7 cm. Pace angles range from 115.0° to 179.4°, with a mean of 154.8°. See S1 Table for all trackway measurements.

### True tracks vs. undertracks

Variation in morphology, along with relatively shallow depths and general lack of detail raise the possibility that some the tracks may be undertracks—sediment deformation resulting from track formation in an overlying sediment layer. One of the benefits of examining this freshly exhumed track surface is that modern weathering is not a complicating factor in interpreting track morphology. The track surface appears to be one extensive planar surface and has no obvious breaks, islands (*sensu* [14]) of overlaying strata, or fenster-like openings revealing underlying strata. In the case of the morphotype 1 tracks, both trackways could be followed directly to the mine’s high wall, where the tracks doubtlessly continued on the bedding-plane surface below the overburden. Examination of the intersection of the bedding plane and the high wall revealed that the track-bearing surface is overlain immediately by the green marine shale. The depositional environment of the green mudstone likely precludes track formation in this bed. Also, although we did not observe it in association with the tridactyl tracks, sauropod tracks in this bed at the Briar Site were noted to be draped by laminae of the overlying green mudstone, which was used as evidence that the tracks are true tracks [2].

**Fig 7:**
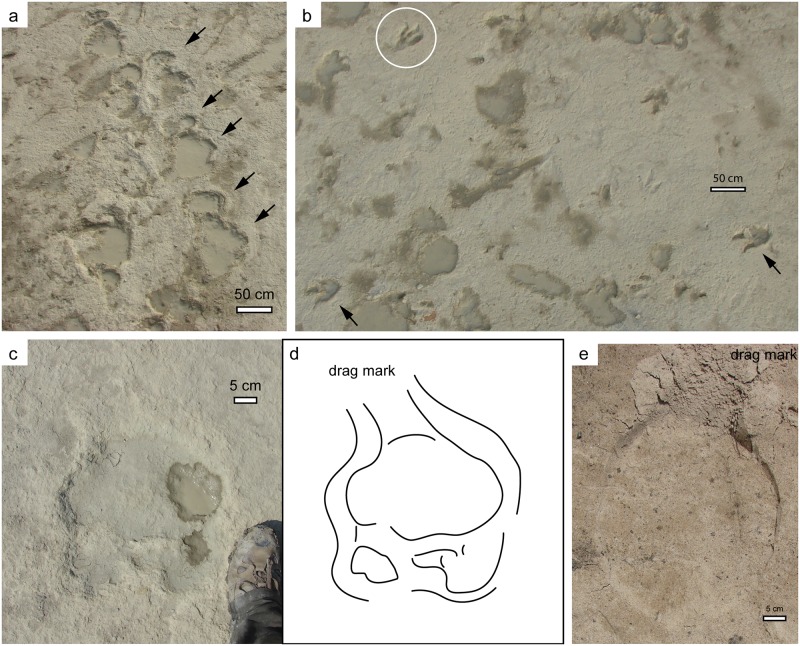
Track surface features indicative of true tracks. (a) Displacement rims around sauropod tracks (arrows); (b) Displacement rims around tridactyl tracks (arrows), note circled track that shows a distorted (abnormally wide) digit IV, likely the result of substrate consistency or behavior; (c-d) Photograph and line drawing interpretation of sauropod manus track with toe drag; (e) African elephant (*Loxodonta africana*) manus track with toe drag, note similarity to feature illustrated in parts c-d.

Features of the tracks themselves can provide additional clues about whether the tracks are true tracks or undertracks. Typically, finely preserved details, including skin impressions, digital pads, claw impressions, drag marks, and expulsion rims are cited as evidence of true tracks [8,14,25,26]. Although there is a general lack of detail in many of the tracks, there are several examples of preserved phalangeal pads and claw marks (Fig 6f, 6g and 6i). Expulsion rims and drag marks are also present in some tracks (Fig 7). One sauropod manus track, in particular, shows a notable example of a toe drag; this is very similar to toe drags made by modern elephants [27], when moving their feet forward out of a track without lifting their feet up high enough to avoid contacting the track shaft wall or expulsion rim (Fig 7c–7e); this feature could only exist in association with a true track.

Neoichnological experiments and modeling aimed at generating undertracks also suggest that a stratified substrate is necessary to cause splitting along a lower, deformed (undertracked) bedding plane [28,29]. The track-bearing bed at the site, however, is massive to thickly bedded and we see no evidence to suggest that a previously overlying bed was evenly eroded before deposition of the green mudstone.

**Fig 8:**
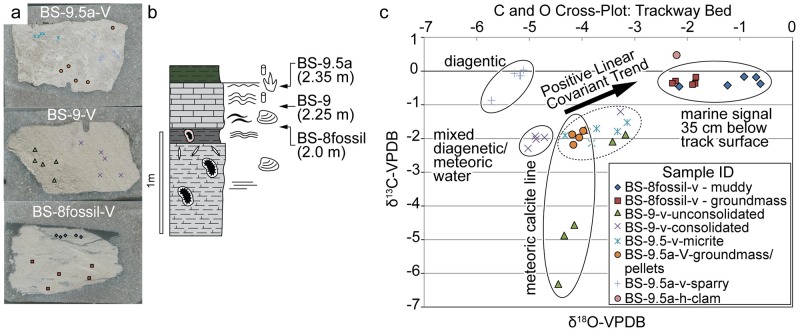
Isotopic composition of the track-bearing bed. (a) Scan of sample billets and sample locations. (b) Stratigraphic locations of samples BS-8 to BS-9.5. (c) A C-O cross-plot of C and O-isotopes values relative to Vienna Pee Dee Belemnite (VPDB). Samples in the upper right of the cross-plot represent a marine signal 35 cm below the surface. Samples 10 cm below the surface (BS-9) show a clear meteoric calcite line, trends common during meteoric water diagenesis suggesting aerial exposure. Samples at the surface (BS-9.5) show a clear positive linear co-variant trend common during evaporative enrichment; data from [30].

Finally, a preliminary stable isotope study [30] analyzed C and O-isotopes of different calcite fabrics from the track-bearing unit and surface (Fig 8). Clear meteoric calcite lines (MCLs) that extend 10 cm below the surface [31,32] and positive linear co-variant trends (PLCTs) at the surface [31,32] are observed in the data. Also observed (30 cm below the surface) is a clear marine signal (positive δ^18^O and δ^13^C). MCLs or trends in C-O isotope space in which C-isotopes are variable and O-isotopes are invariant represent meteoric diagenesis in which O is controlled by the average δ^18^O of meteoric groundwater and δ^13^C represents mixing between atmospheric and respired CO_2_. PLCTs are similar to MCLs, however δ^18^O has a positive trend due to evaporative enrichment of ^18^O, with the slope of that trend being controlled by the intensity of evaporation. These trends are most common in pedogenic carbonates and surface exposures and are less likely to occur in undertracks [33]. If the track surface represents undertracks, it would most likely have a marine cement signal rather than a meteoric diagenetic signal. Based on the abundance of the evidence, we interpret most, if not all, of the tracks as true tracks.

### Influence of substrate on preservation and morphology

Tracks exhibit a wide range of morphologies, even within the same trackway, suggesting that variations in substrate consistency strongly influenced track preservation at the site. A qualitative comparison of morphotype 1 and morphotype 2 tracks shows that, although morphotype 1 tracks are generally larger than morphotype 2 tracks, several characteristics are very similar (e.g., detached digit II, relative digit lengths, phalangeal pads, pointed metatarsal impressions, claw impressions, and total divarication). A previously published preliminary plot of length versus width shows one continuous trend with a consistent slope [3], which suggests that the apparent two morphotypes are actually different-sized and differentially preserved tracks from the same population, i.e., from different sized individuals of the same species [34].

It is our contention that the tridactyl tracks are from a single species of trackmaker and appear as two morphotypes because of differences in animal size and substrate consistency. Much neoichnological experimentation and modeling have focused on the effects of sediment properties on track appearance and preservation [27,28,35–37] and here we apply a simple method to assess differences in substrate conditions at the time of track formation.

Because the cohesion of the substrate determines to what degree the sediment directly adjacent to a footprint deforms during and after the trackmaker takes a step [35,37,38], the slope of the track (shaft [39]) wall should be a proxy for sediment consistency. The slope of the track wall will define the difference between the true track dimensions (Fig 2a) and the apparent track dimensions (Fig 2a) [22]. As long as the tracks do not exhibit overhanging walls [22], a greater disparity between true and apparent dimensions indicates a greater slope and a higher amount of deformation of the surrounding sediment. A greater slope angle can indicate either less cohesion in the case of a dry unconsolidated substrate [36,37] or it can indicate greater cohesion in a moist, but not saturated, substrate [35,36]. In the case of the tridactyl tracks, a moist substrate is most consistent with the interpreted depositional environment.

**Fig 9:**
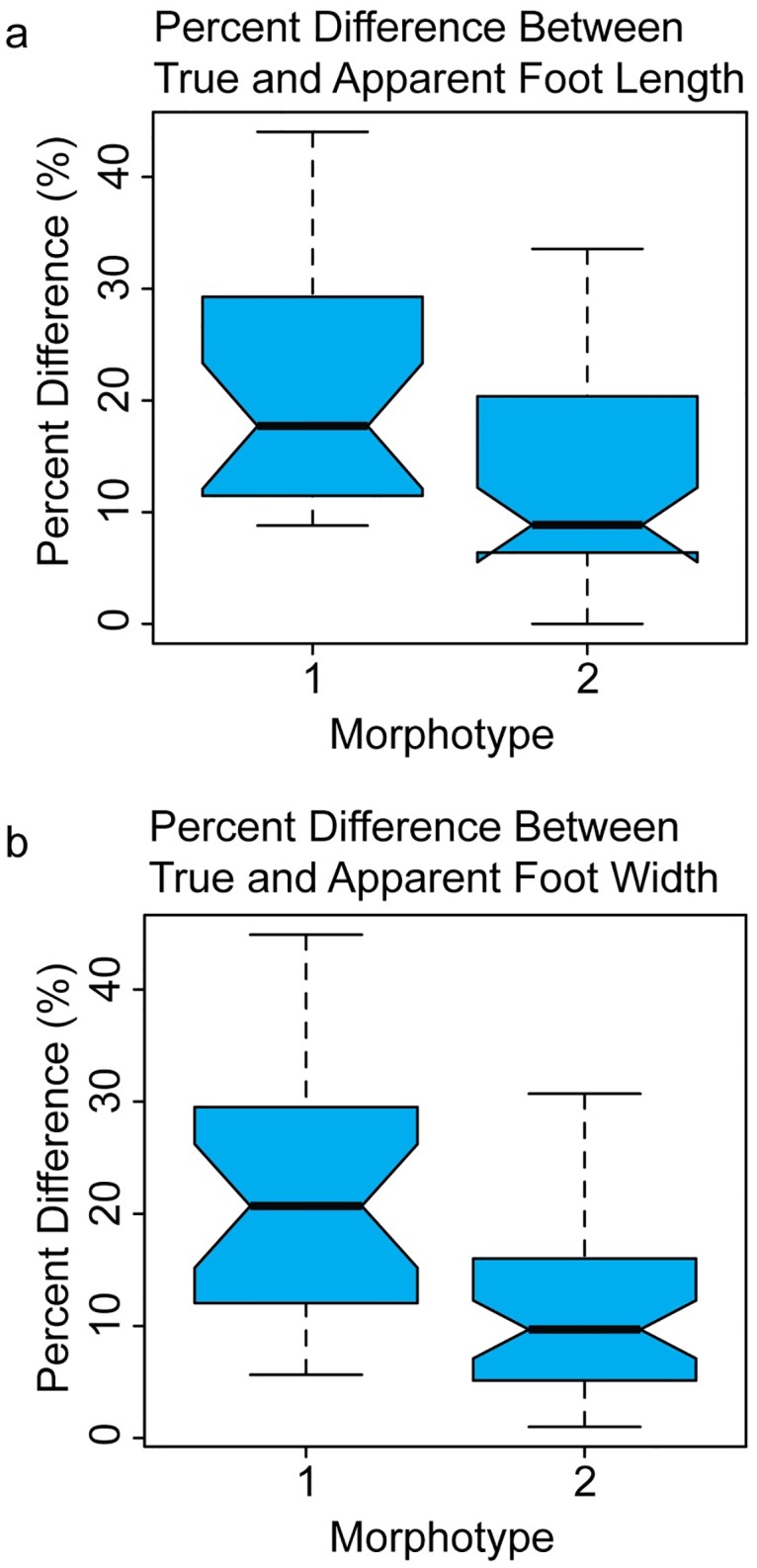
Comparison between tridactyl track morphotypes. (a) Notched box plots of percent difference between true and apparent foot length showing a significant difference between morphotypes 1 and 2. (b) Notched box plots of percent difference between true and apparent foot width showing a significant difference between morphotypes 1 and 2.

Comparison of the percent difference between true and apparent track length and width (S2 Table) shows that morphotype 1 has significantly larger differences than morphotype 2 (Fig 9). Morphotype 1 tracks are also shallower than morphotype 2 tracks, although this relationship is not statistically significant. As a result, we interpret the consistently wide digits of morphotype 1 tracks as the result of the pads of the feet expanding outward due to stepping on a firm substrate [40]. Outward expansion of phalangeal pads is observed in extant birds and is inferred to have occurred in dinosaur feet [40]. Sediment firmness may be controlled by a number of factors, but bulk density by far has the greatest influence over the depth of foot penetration into a substrate [27]. Thus, a greater bulk density results in shallower tracks with wider apparent dimensions. Bulk density approaches its maximum for a given sediment at an optimum moisture content, which likely characterizes the sediment at the southern end of the tracksite where the shallower morphotype 1 tracks are found. Interestingly, this part of the tracksite also contains well-preserved manus-only sauropod tracks (S1 Fig), which are possible to generate in sediment characterized by high shear strength [41]. High shear strength is correlated with high bulk density in tidal flat sediments [42].

### Theropod vs. ornithopod

The two major groups of dinosaurs responsible for making tridactyl tracks are theropods and ornithopods [43]. Distinguishing between their tracks can be challenging, but a number of qualitative features and quantitative metrics have been proposed to differentiate between the two groups [20,43,44]. Qualitatively, the tridactyl tracks described here contain a large number of features that are considered characteristic of theropods, including examples of narrow and tapering claw impressions, clear phalangeal pad impressions, slender digits, long and sinuous digit III, and digit II separate from digits III and IV [44] (Fig 6).

**Fig 10:**
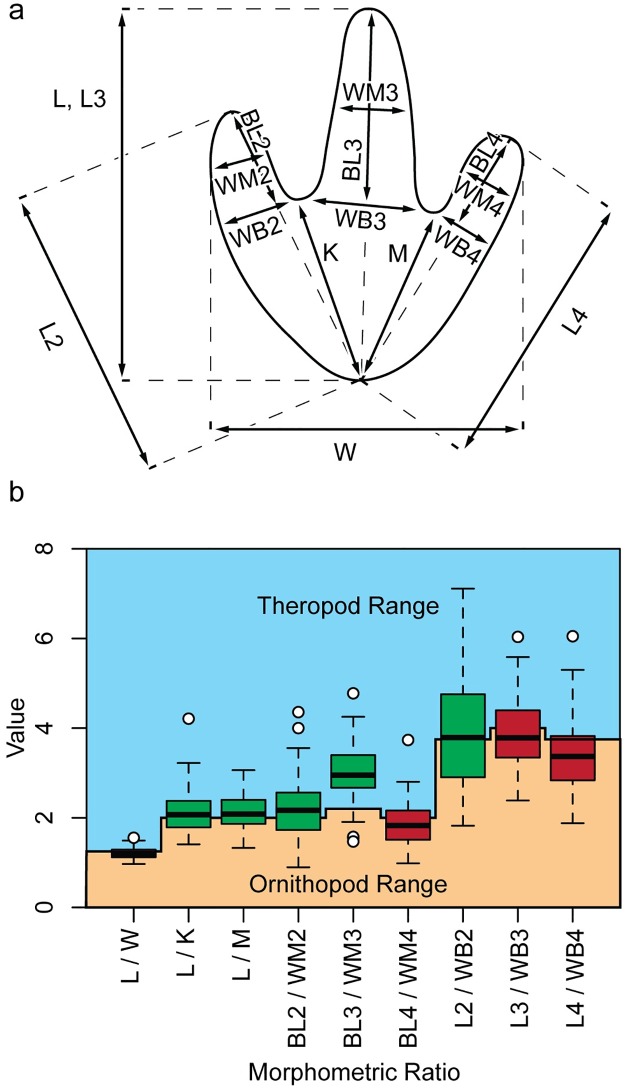
Box plots showing the range of morphometric ratios from De Queen tridactyl tracks relative to thresholds for differentiating between theropod and ornithopod dinosaur tracks. Green boxes indicate medians fall in the theropod range; red boxes indicate median falls within the ornithopod range; L = length, W = width, K = length between heel and hypex between digits II and III, M = length between heel and hypex between digits III and IV, BL2 = digit II basal length, WM2 = middle digit width of digit II, BL3 = digit III basal length, WM3 = middle digit width of digit III, BL4 = digit IV basal length, WM4 = middle digit width of digit IV, L2 = digit II length, WB2 = basal digit II width, L3 = digit III length, WB3 = basal digit III width, L4 = digit IV length, WB4 = basal digit IV width. Five medians fall within the theropod range, three fall within the ornithopod range, and one falls on the threshold.

We also apply the quantitative method of [20], who calculate nine ratios between fifteen track parameters (Fig 10a). Results of measurements from all tridactyl trackways at the site (Fig 10b; S3 Table) show that many of the ratios plot very close to the boundary between theropod and ornithopod. The medians of five ratios plot within the theropod zone of the graph and three fall within the ornithopod range, but note that those three are all ratios involving digit 3 and 4 measurements, which show distortion in some tracks, likely from substrate consistency or behavior (e.g., Fig 7b). Based on the abundance of evidence, we attribute all tridactyl tracks at the site to theropods.

### Theropod trackmaker identity

It is natural to compare the De Queen theropod tracks to the theropod tracks in the equivalent Glen Rose Formation of Texas, just as previous comparisons have been made between De Queen and Glen Rose sauropod tracks [2]. We used published datasets [45,46] that contain the mean, minimum, and maximum measurements for the footprint length, pace, stride, and pace angulation of many (n = 126) of the Glen Rose trackways. The data (S1 Table) do not conform to the assumptions of parametric statistical tests so we performed Wilcoxon rank-sum tests to compare mean footprint length, mean pace, mean stride, and mean pace angulation between De Queen and Glen Rose trackways. Results showed no significant differences between any of the trackway metrics from the two populations (Table 1) so we conclude that the De Queen and Glen Rose trackways are attributable to the same track-making taxon.

**Table 1:** Results of Wilcoxon rank-sum tests comparing De Queen and Glen Rose tridactyl trackway data.

	De Queen median	Glen Rose median	W	p	r
**Mean footprint length (mm)**	460.0	488.7	403	0.3949	-0.074
**Mean pace (cm)**	143.0	146.4	440	0.6416	-0.043
**Mean stride (cm)**	281.4	291.0	331	0.6981	-0.041
**Mean pace angulation** (°)	165.5	158.7	196	0.4219	-0.118

Note that all tests yielded nonsignificant results (p > 0.05) so it is reasonable to conclude that the De Queen and Glen Rose trackways represent the same trackmaking taxon.

The large sizes of many of these tracks limit potential trackmaking species. The large size of the Glen Rose tracks corresponds to the only large theropod known from skeletal material in nearby Lower Cretaceous rocks, *Acrocanthosaurus*; the relationship between track dimensions and pedal measurements from *Acrocanthosaurus* skeletal material has already been established [47]. We likewise attribute the De Queen tracks to *Acrocanthosaurus*, especially given that skeletal remains have been recovered from the Aptian-Albian Antlers Formation 90 km away in Idabel, Oklahoma [48] and from the mine site itself [49].

### Size and speed

Despite a number of allometric and morphometric equations for estimation of hip height of large theropods [43], [50] found that the simple equation 4*FL (where FL = footprint length) provides the most accurate estimate. Using mean true footprint length, we calculate hip heights of the theropod trackmakers to range between 1.5 and 2.2 m (S4 Table). Using these values and mean stride lengths for each trackway, we calculate the speeds of the theropods to have ranged between 1.87 and 2.54 m/s using the speed equation of [51]. Corresponding relative stride lengths range between 1.39 m and 1.78 m, qualifying all trackways as representing walking gaits [43]. Inward rotation of the tracks also agrees with an interpretation of walking theropods [52].

### Trackway orientations

**Fig 11:**
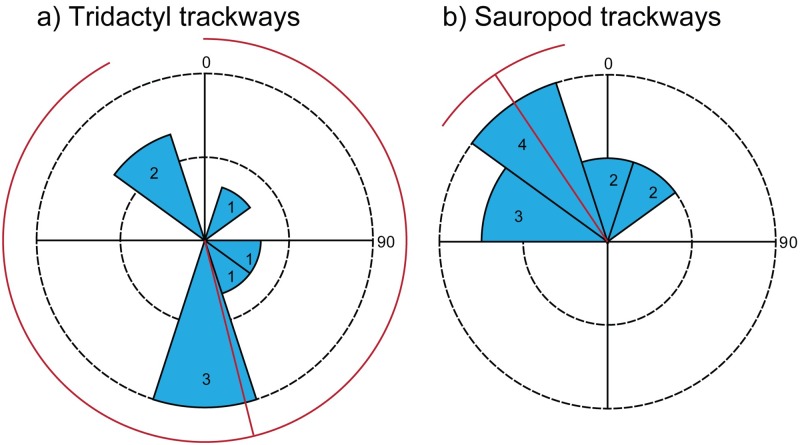
Rose diagrams showing trackway orientations. (a) Theropod trackway orientations with circular mean and 95% confidence interval (red lines). (b) Sauropod trackway orientations with circular mean and 95% confidence interval (red lines).

Rose diagrams (Fig 11; S5 Table) generated with PAST [53] show that the theropod trackways do not show any preferential orientation. Sauropod trackways, on the other hand, are dominantly oriented to the NW with a circular mean of 326°. Note, however, that these data should be considered tentative because of the small trackway sample size. There is no direct evidence of any interactions between the sauropods and the theropods nor is there overlap of their tracks so we cannot make any definite conclusions regarding the relative timing of these animals’ activity. One prevalent pattern is a pair of sinuous sauropod trackways that parallel each other for > 50 m (S1 Fig, trackways Q1 and Q4), suggesting that the two trackmaking individuals were walking together at the same time. Also, the longest of these two trackways contains very closely spaced tracks that do not conform to the majority of strides in the trackway (S1 Fig, trackway Q8), suggesting a possible third individual that was following or being followed.

## Conclusions

LIDAR is being increasingly used to characterize dinosaur tracksites, especially for preservation of data from endangered sites. We employed this tool, in combination with photography and plaster casting to preserve and characterize an aerially extensive trackway situated in an active quarry within the De Queen Formation that contains the first tridactyl trackways known from the state of Arkansas. The tracksite contains sauropod and large theropod trackways preserved in a single bedding plane representative of a broad, carbonate mudflat setting.

We use a simple approach to interpreting relative substrate consistency that uses the difference between the true track dimensions and the apparent track dimensions as a proxy for track wall deformation. Results show that shallower tracks with greater track wall deformation constitute one apparent morphotype that differs in appearance from the rest of the tridactyl tracks. Upon closer examination, however, all tridactyl tracks appear to be from different sized individuals of the same track-making species and the morphological differences are the result of substrate variation. In particular, the wider, more robust tridactyl tracks represent outward protrusion of the fleshy parts of the feet while pressing against firm sediment with high bulk density and high shear strength. This interpretation is corroborated by associated manus-only sauropod tracks.

A number of qualitative properties and quantitative metrics point to a large theropod dinosaur as the likely trackmaker. Statistical comparison to tridactyl tracks from the equivalent Glen Rose Formation of Texas cannot reject the null hypothesis that the tracks constitute two different populations. We therefore interpret the De Queen theropod trackmaker as *Acrocanthosaurus*, the animal inferred to have made the Glen Rose tracks. *Acrocanthosaurus* is a large theropod dinosaur known from skeletal remains from the mine and equivalent Lower Cretaceous units, is the correct size, and has the correct pedal anatomy to have made the De Queen tracks. These trackways, thus, extend the known range of *Acrocanthosaurus* eastward by some 90 km and represent its easternmost known occurrence west of the Atlantic Coastal Plain.

In addition to allowing detailed ichnological analysis, the LIDAR data preserve the tracksite digitally for future study. Shortly after our data collection campaign the track-bearing layer was removed with explosives to reach the minable bedded gypsum. These data not only allow conservation of the scientific value of the now destroyed site, but they also allow unlimited access to researchers and the public alike. In fact, interactive hillshade relief images derived from the LIDAR data are currently available online (http://trackways.cast.uark.edu/index.html). Through the use of LIDAR, we have been able to optimize the balance between scientific, cultural, and economic interests within a shared region.

## Supporting information

S1 Fig Tracksite map. (left) Photomosaic of track surface. (middle) Hillshade relief image from LIDAR point cloud, lighting from 45°. (right) Trackway map with color-coded interpretations of theropod and sauropod trackways.
(PDF)

S1 Table Tridactyl trackway measurements. (XLSX)

S2 Table Tridactyl track measurements. See Fig 2 for additional explanation.
(XLSX)

S3 Table Tridactyl track parameters for determine theropod vs. ornithopod origin. See Fig 9a for illustration of track parameters.
(XLSX)

S4 Table Estimates of hip heights and speeds of tridactyl trackmakers. Hip heights are calculated as FLT multiplied by 4 [50]; speeds are calculated according to [51].
(XLSX)

S5 Table Trackway orientations. Measured by protractor on the hillshade relief map generated from LIDAR data.
(XLSX)

## References

[1] Bird R. Thunder in his footsteps. Nat Hist. 1939;43: 254–261.

[2] Pittman J, Gillette D. The Briar Site: a new sauropod dinosaur tracksite in Lower Cretaceous beds of Arkansas, USA In: Gillette D, Lockley M, editors. Dinosaur Tracks and Traces. Cambridge: Cambridge University Press; 1989 pp. 313–332.

[3] Shell R, Boss SK. Morphometric analysis of dinosaur tracks from southwest Arkansas. J Arkansas Acedemy Sci. 2013;67: 121–130.

[4] Breithaupt BH, Matthews NA, Noble TA. An integrated approach to three-dimensional data collection at dinosaur tracksites in the Rocky Mountain West. Ichnos. 2004;11: 11–26.

[5] Bates KT, Rarity F, Manning PL, Hodgetts D, Vila B, Oms O, et al High-resolution LiDAR and photogrammetric survey of the Fumanya dinosaur tracksites (Catalonia): implications for the conservation and interpretation of geological heritage sites. J Geol Soc London. 2008;165: 115–127.

[6] Bates KT, Manning PL, Vila B, Hodgetts D. Three-dimensional modelling and analysis of dinosaur trackways. Palaeontology. 2008;51: 999–1010.

[7] Vila B, Oms O, Galobart À, Bates KT, Egerton VM, Manning PL. Dynamic similarity in titanosaur sauropods: ichnological evidence from the Fumanya dinosaur tracksite (southern Pyrenees). PLoS One. 2013;8: 1–9. doi: 10.1371/journal.pone.0057408 23451221 10.1371/journal.pone.0057408 PMC3581443

[8] Marty D. Sedimentology, taphonomy, and ichnology of Late Jurassic dinosaur tracks from the Jura carbonate platform (Chevenez—Combe Ronde tracksite, NW Switzerland): insights into the tidal-flat palaeoenvironment and dinosaur diversity, locomotion, and palaeoecology. GeoFocus. 2008;21: 1–278.

[9] Falkingham PL, Bates KT, Farlow JO, Szeliski R, Crompton R. Historical photogrammetry: Bird’s Paluxy River dinosaur chase sequence digitally reconstructed as it was prior to excavation 70 years ago. PLoS One. 2014;9: 1–5. doi: 10.1371/journal.pone.0093247 24695537 10.1371/journal.pone.0093247 PMC3973721

[10] Thomas DA, Farlow JO. Tracking a dinosaur attack: the efforts of a sculptor and a paleontologist reveal details of a 100-million-year-old skirmish. Sci Am. 1997; 74–79.9198897

[11] Farlow JO, Pittman JG, Hawthorne JM. *Brontopodus birdi*, Lower Cretaceous sauropod footprints form the U.S. Gulf Coastal Plain In: Gillette DD, Lockley MG, editors. Dinosaur Tracks and Traces. Cambridge: Cambridge University Press; 1989 pp. 371–394.

[12] Bates KT, Falkingham PL, Hodgetts D, Farlow JO, Breithaupt BH, O’Brien M, et al Digital imaging and public engagement in palaeontology. Geol Today. 2009;25: 134–139. doi: 10.1111/j.1365-2451.2009.00714.x

[13] Farlow JO, O’Brien M, Kuban GJ, Dattilo BF, Bates KT, Falkingham PL, et al Dinosaur Tracksites of the Paluxy River Valley (Glen Rose Formation, Lower Cretaceous), Dinosaur Valley State Park, Somervell County, Texas. Actas V Jornadas Int sobre Paleontol Dinosaur y su Entorno, Salas los Infantes, Burgos. 2012; 41–69.

[14] Castanera D, Vila B, Razzolini NL, Falkingham PL, Canudo JI, Manning PL, et al Manus track preservation bias as a key factor for assessing trackmaker identity and quadrupedalism in basal ornithopods. PLoS One. 2013;8: 1–13. doi: 10.1371/journal.pone.0054177 23349817 10.1371/journal.pone.0054177 PMC3551957

[15] Colbert EH, Merrilees D. Cretaceous dinosaur footprints from Western Australia. J R Soc West Aust. 1967;50: 21–25.

[16] Romilio A, Hacker JM, Zlot R, Poropat G, Bosse M, Salisbury SW. A multidisciplinary approach to digital mapping of dinosaurian tracksites in the Lower Cretaceous (Valanginian–Barremian) Broome Sandstone of the Dampier Peninsula, Western Australia. PeerJ. 2017; 1–30. doi: 10.7717/peerj.3013 28344899 10.7717/peerj.3013 PMC5363262

[17] Pittman J. Geology of the De Queen Formation of Arkansas. Gulf Coast Assoc Geol Soc Trans. 1984;34: 201–209.

[18] Pittman J. Stratigraphy, lithology, depositional environment, and track type of dinosaur track-bearing beds of the Gulf Coastal Plain In: Gillette D, Lockley MG, editors. Dinosaur Tracks and Traces. Cambridge: Cambridge University Press; 1989 pp. 135–153.

[19] Leonardi G, editor. Glossary and manual of tetrapod palaeoichnology. Brasil: Departamento Nacional da Produção Mineral; 1987.

[20] Moratalla JJ, Sanz JL, Jimenez S. Multivariate analysis on Lower Cretaceous dinosaur footprints: discrimination between ornithopods and theropods. Geobios. 1988;21: 395–408. doi: 10.1016/S0016-6995(88)80042-1

[21] Olsen PE, Smith JB, McDonald NG. Type material of the type species of the classic theropod footpint genera *Eubrontes*, *Anchisauripus*, and *Grallator* (Early Jurassic, Hartford and Deerfield Basins, Connecticut and Massachusetts, U.S.A. J Vertebr Paleontol. 1998;18: 586–601.

[22] Lockley MG, Schulp AS, Meyer C, Leonardi G, Mamani DK. Titanosaurid trackways from the Upper Cretaceous of Bolivia: evidence for large manus, wide-gauge locomotion and gregarious behavior. Cretac Res. 2002;23: 383–400.

[23] Hasiotis ST, Platt BF, Hembree DI, Everhart MJ. The Trace-Fossil Record of Vertebrates In: Miller WI, editor. Trace Fossils: Concepts, Problems, Prospects. Amsterdam: Elsevier; 2006 pp. 196–218.

[24] Goodwin MB, Chaney DS. Molding, casting, and painting In: Leiggi P, May P, editors. Vertebrate Paleontological Techniques. Cambridge: Cambridge University Press; 1995 pp. 235–284.

[25] Mezga A, Tesovic BC, Bajraktarevic Z. First record of dinosaurs in the Late Jurassic of the Adriatic-Dinaridic carbonate platform (Croatia). Palaios. 2007;22: 188–199.

[26] Fujita M, Lee Y-N, Azuma Y, Li D. Unusual tridactyl trackways with tail traces from the Lower Cretaceous Hekou Group, Gansu Province, China. Palaios. 2012;27: 560–570.

[27] Platt B, Hasiotis S, Hirmas D. Empirical determination of physical controls on megafaunal footprint formation through neoichnological experiments with elephants. Palaios. 2012;27: 725–737.

[28] Milàn J, Bromley RG. True tracks, undertracks and eroded tracks, experimental work with tetrapod tracks in laboratory and field. Palaeogeogr Palaeoclimatol Palaeoecol. 2006;231: 253–264.

[29] Gatesy SM, Ellis RG. Beyond surfaces: a particle-based perspective on track formation In: Falkingham PL, Marty D, Richter A, editors. Dinosaur Tracks: The Next Steps. Bloomington, Indiana: Indiana Univerity Press; 2016 pp. 82–91.

[30] Platt B, Shell R, Suarez C, Boss S, Williamson M. The first theropod trackways from the Lower Cretaceous (Albian) De Queen Formation, Southwest Arkansas. J Vertebr Paleontol Suppl. 2013; 192.

[31] Ufnar DF, Ludvigson GA, González L, Gröcke DR. Precipitation rates and atmospheric heat transport during the Cenomanian greenhouse warming in North America: Estimates from a stable isotope mass-balance model. Palaeogeogr Palaeoclimatol Palaeoecol. 2008;266: 28–38. doi: 10.1016/j.palaeo.2008.03.033

[32] Suarez MB, González LA, Ludvigson GA, Vega FJ, Alvarado-Ortega J. Isotopic composition of low-latitude paleoprecipitation during the Early Cretaceous. Bull Geol Soc Am. 2009;121: 1584–1595. doi: 10.1130/B26453.1

[33] Phillips PLJ, Ludvigson GA, Joeckel RM, González LA, Brenner RL, Witzke BJ. Sequence stratigraphic controls on synsedimentary cementation and preservation of dinosaur tracks: example from the lower Cretaceous (Upper Albian) Dakota Formation, southeastern Nebraska, U.S.A. Palaeogeogr Palaeoclimatol Palaeoecol. 2007;246: 367–389.

[34] Lockley MG. Dinosaur ontogeny and population structure: interpretations and speculations based on fossil footprints In: Carpenter K, Hirsch KF, Horner JR, editors. Dinosaur Eggs and Babies. Cambridge: Cambridge University Press; 1994 pp. 347–365.

[35] Falkingham PL, Margetts L, Manning PL. Fossil vertebrate tracks as paleopenetrometers: confounding effects of foot morphology. Palaios. 2010;25: 356–360.

[36] Jackson SJ, Whyte MA, Romano M. Range of experimental dinosaur (*Hypsilophodon foxii*) footprints due to variation in sand consistency: how wet was the track? Ichnos. 2010;17: 197–214. doi: 10.1080/10420940.2010.510026

[37] Falk AR, Hasiotis ST, Gong E, Lim J-D, Brewer ED. A new experimental setup for studying avian neoichnology and the effects of grain size and moisture content on tracks: trials using the domestic chicken (*Gallus gallus*). Palaios. 2017;32: 689–707. doi: 10.2110/palo.2017.022

[38] Milàn J. Variations in the morphology of emu (*Dromaius novaehollandiae*) tracks reflecting differences in walking pattern and substrate consistency: ichnotaxonomic implications. Palaeontology. 2006;49: 405–420.

[39] Allen JRL. Subfossil mammalian tracks (Flandrian) in the Severn Estuary, S. W. Britain: mechanics of formation, preservation and distribution. Philos Trans R Soc London B. 1997;352: 481–518.

[40] Gatesy SM. Skin impressions of Triassic theropods as records of foot movement. Bull Museum Comp Zool. 2001;156: 137–149.

[41] Falkingham PL, Bates KT, Margetts L, Manning PL. Simulating sauropod manus-only trackway formation using finite-element analysis. Biol Lett. 2011;7: 142–145. doi: 10.1098/rsbl.2010.0403 20591856 10.1098/rsbl.2010.0403 PMC3030862

[42] Amos C, Van Wagoner N, Daborn G. The influence of subaerial exposure on the bulk properties of fine-grained intertidal sediment from Minas Basin, Bay of Fundy. Estuar Coast Shelf Sci. 1988;27: 1–13.

[43] Thulborn T. Dinosaur Tracks. London: Chapman and Hall; 1990.

[44] Dalla Vecchia FM, Tarlao A. New dinosaur track sites in the Albian (Early Cretaceous) of the Istrian peninsula (Croatia)—Part II—Paleontology. Mem di Sci Geol. 2000;52: 227–292.

[45] Farlow JO. Lower Cretaceous dinosaur tracks, Paluxy River Valley, Texas: Field Trip Guidebook, South Central Section, Geological Society of America. Waco: Baylor University; 1987.

[46] Farlow JO, Langston W Jr., Deschner EE, Solis R, Ward W, Kirkland BL, et al Texas Giants: Dinosaurs of the Heritage Museum of the Texas Hill Country. Canyon Lake, TX: The Heritage Museum of the Texas Hill Country; 2006.

[47] Farlow J. *Acrocanthosaurus* and the maker of Comanchean large-theropod footprints In: Tanke DH, Carpenter K, Skrepnick MW, editors. Mesozoic Vertebrate Life. Bloomington: Indiana University Press; 2001 pp. 408–427.

[48] D’Emic MD, Melstrom KM, Eddy DR. Paleobiology and geographic range of the large—bodied Cretaceous theropod dinosaur *Acrocanthosaurus atokensis*. Palaeogeogr Palaeoclimatol Palaeoecol. 2012;333–334: 13–23. doi: 10.1016/j.palaeo.2012.03.003

[49] Suarez C, Frederickson J, Cifelli RL, Nydam RL, Pittman J, Morgan K, et al The vertebrate fauna from the Lower Cretaceous Holly Creek Formation. The first multi-taxa vertebrate assemblage from the Mesozoic of Arkansas, USA. Geol Soc Am Abstr with Programs. 2017;49.

[50] Henderson DM. Footprints, trackways, and hip heights of bipedal dinosaurs—testing hip height predictions with computer models. Ichnos. 2003;10: 99–114.

[51] Alexander RM. Estimates of speeds of dinosaurs. Nature. 1976;261: 129–130. doi: 10.1038/261129a0

[52] Day JJ, Norman DB, Upchurch P, Powell HP. Dinosaur locomotion from a new trackway. Nature. 2002;415: 494–495. doi: 10.1038/415494a 11823849 10.1038/415494a

[53] Ryan PD, Hammer Ø, Harper DA, Paul Ryan DD. PAST: paleontological statistics software package for education and data analysis. Palaeontol Electron. 2001;4: 1–9.

