# Supplementary figures and images for: LIDAR-based characterization and conservation of the first theropod dinosaur trackways from Arkansas, USA
Source: PLoS One. 2018 Jan 2;13(1):e0190527. doi: 10.1371/journal.pone.0190527 (PMC5749850; doi:10.1371/journal.pone.0190527)

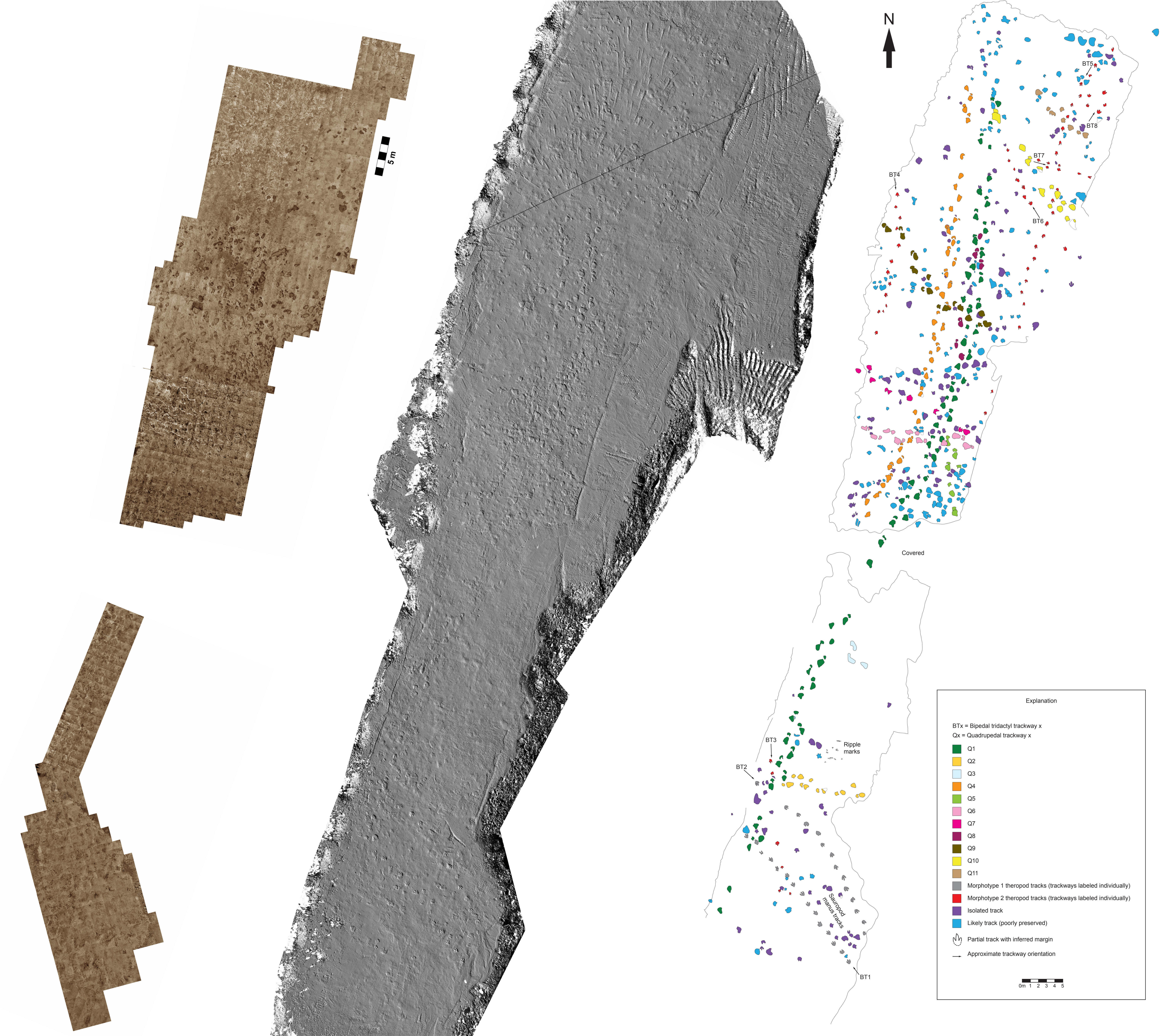

Supplement: S1 Fig — (left) Photomosaic of track surface. (middle) Hillshade relief image from LIDAR point cloud, lighting from 45°. (right) Trackway map with color-coded interpretations of theropod and sauropod trackways. (PDF) [file pone.0190527.s001.pdf]
